# Supplementary figures and images for: Dynamic changes in chromosome and nuclear architecture during maturation of normal and ALS C9orf72 motor neurons
Source: bioRxiv. 2025 Sep 22:2025.09.22.677835. Preprint. [Version 1] doi: 10.1101/2025.09.22.677835 (PMC12485917; doi:10.1101/2025.09.22.677835)

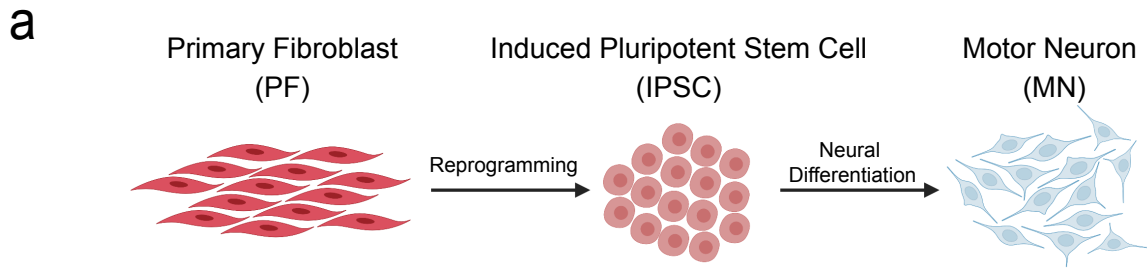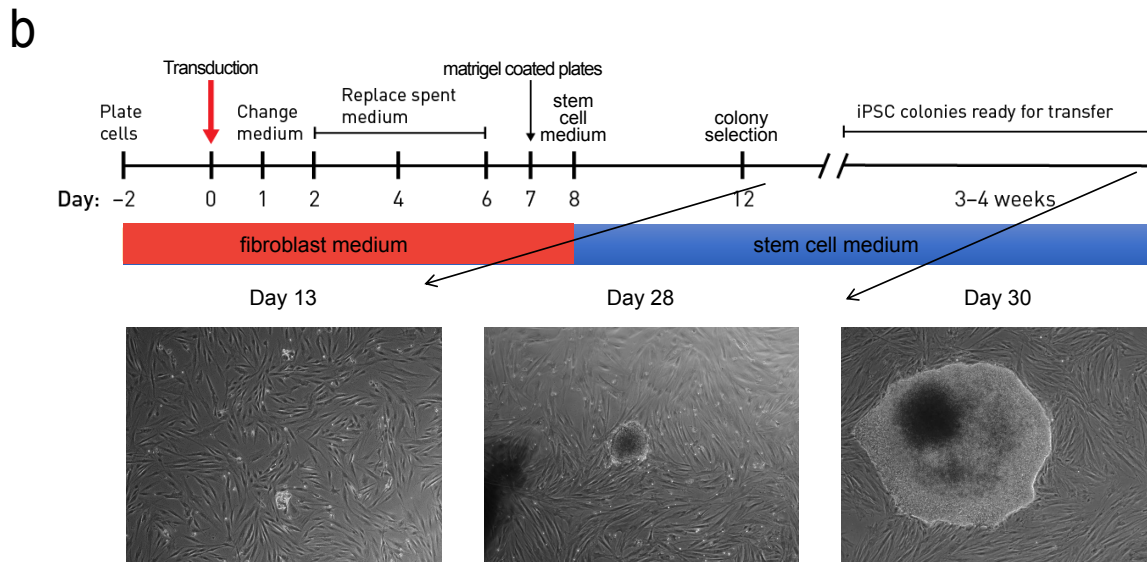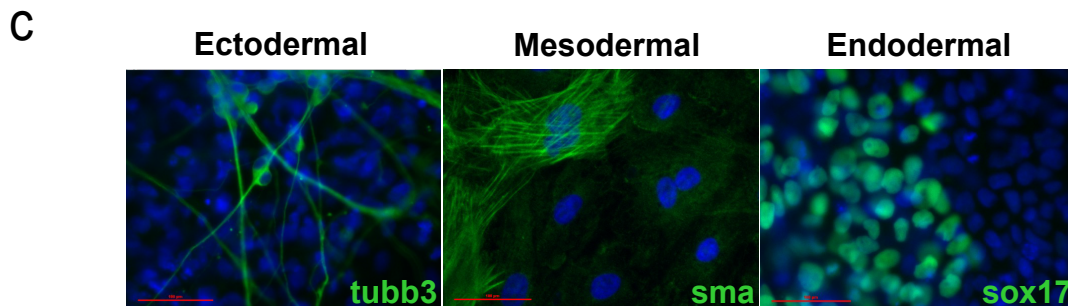

Supplement: Supplement 1 [file media-1.pdf]

**Figure S2**

**a**

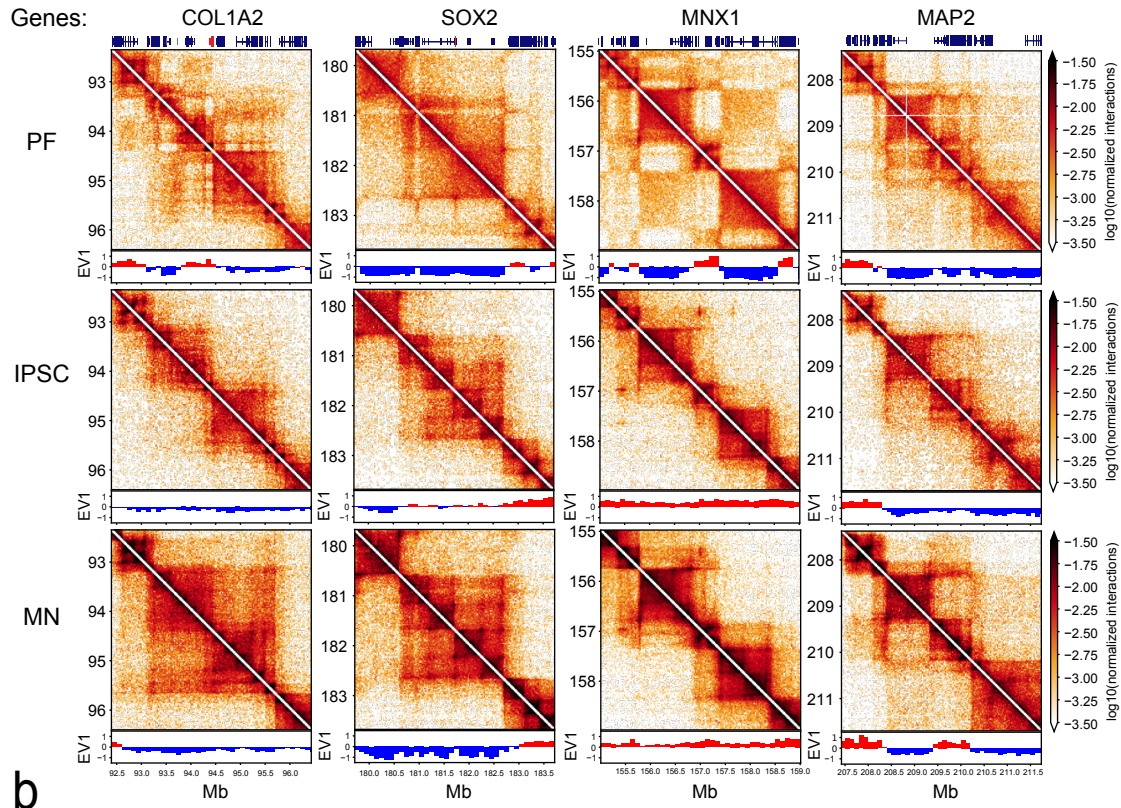

**b**

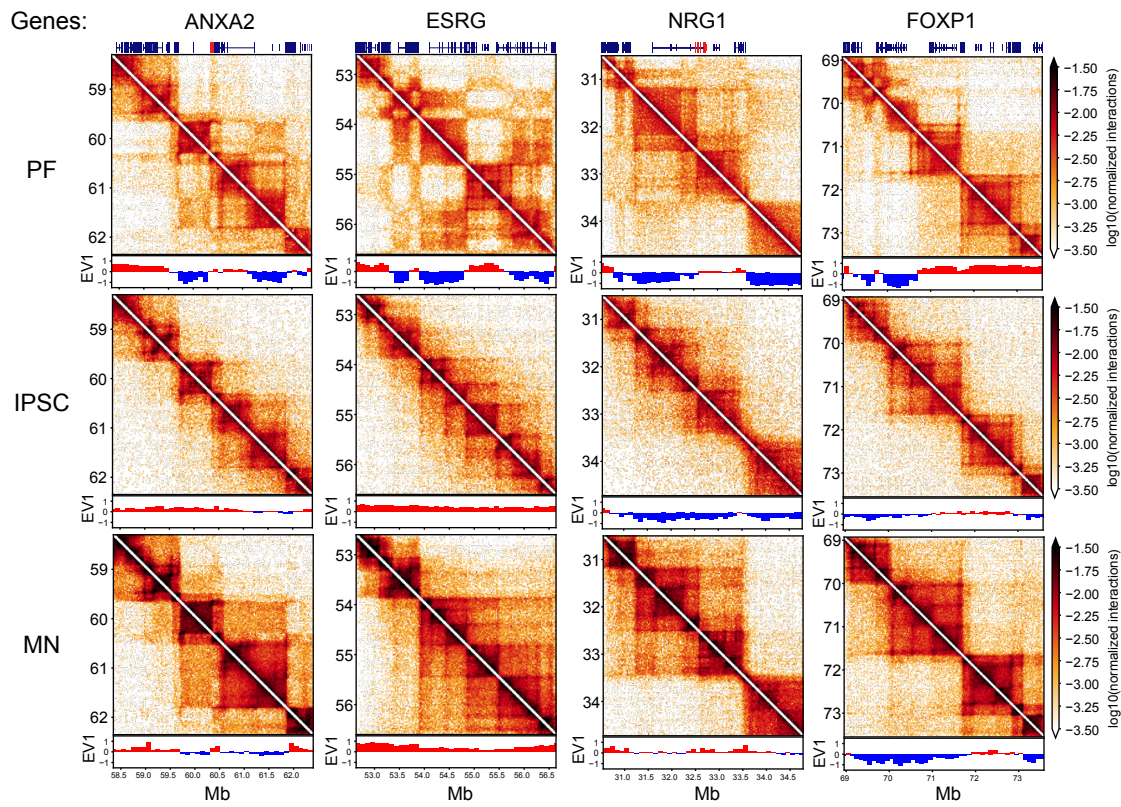

Supplement: Supplement 2 [file media-2.pdf]

# Figure S2

**C**

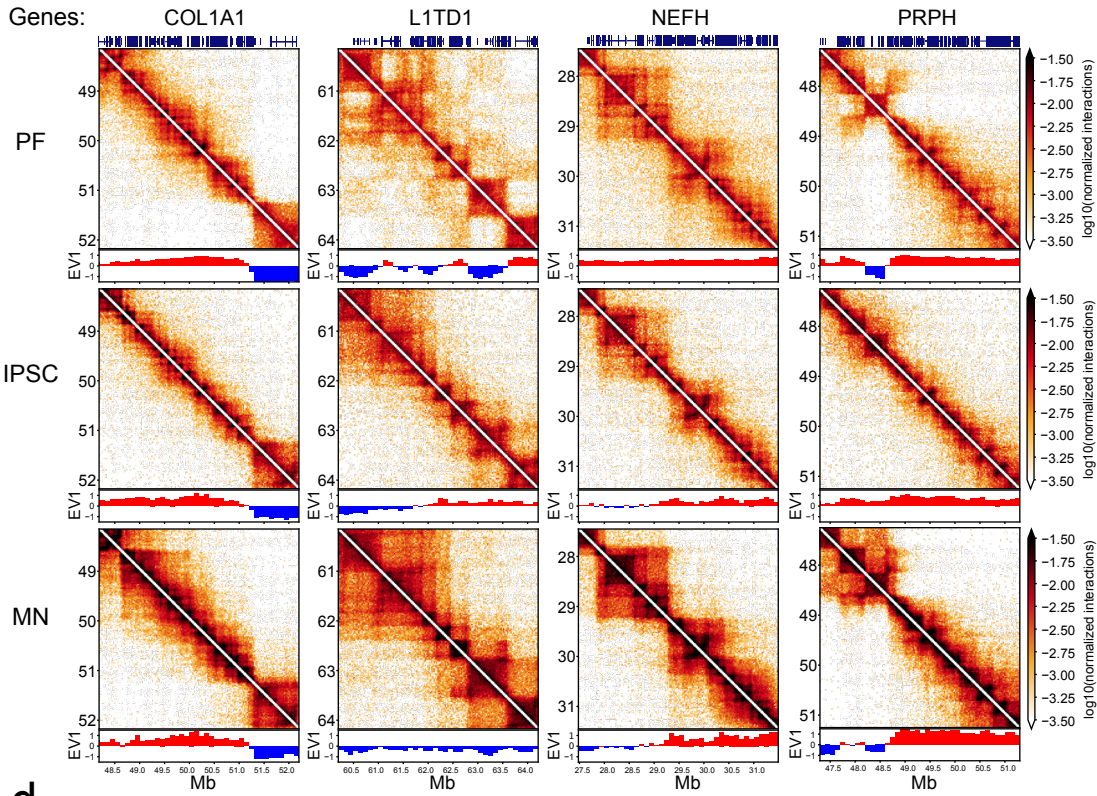

**d**

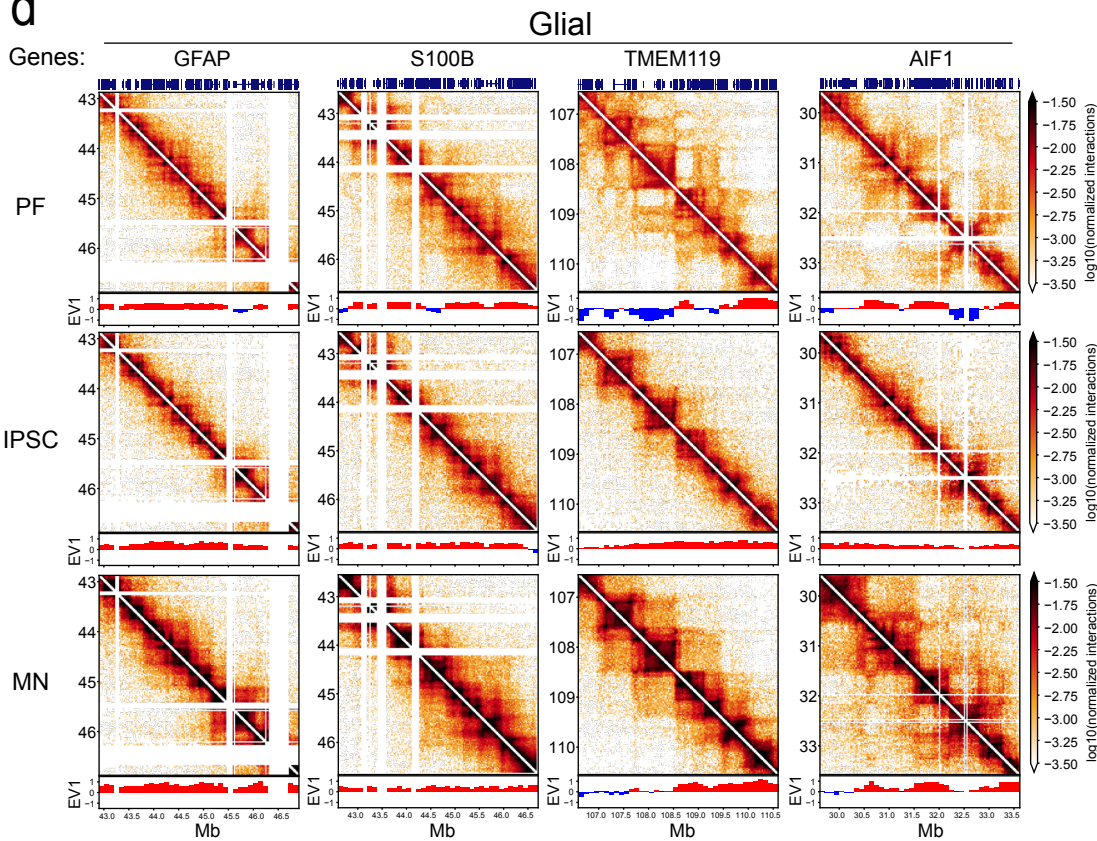

Supplement: Supplement 3 [file media-3.pdf]

a

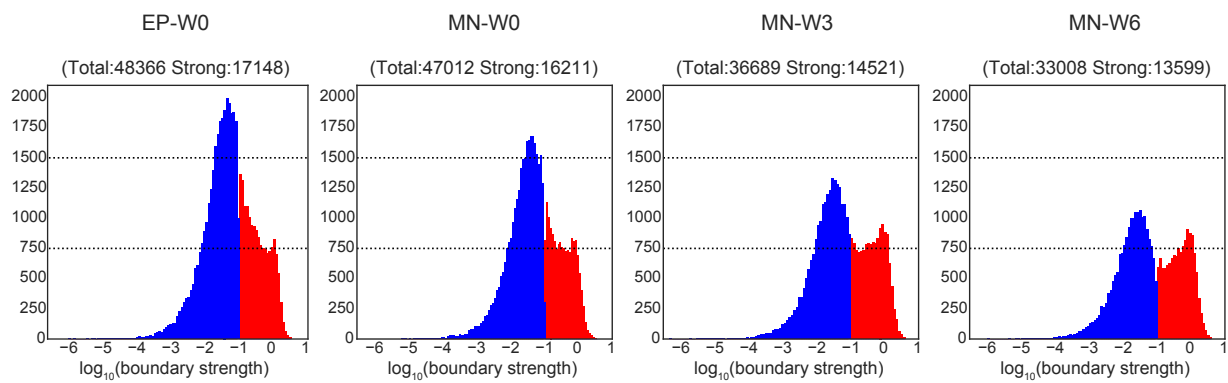

Supplement: Supplement 4 [file media-4.pdf]

**Figure S7**

**a**

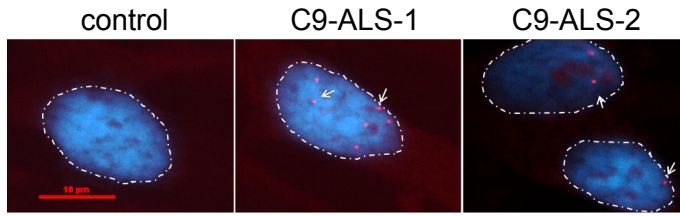

**b**

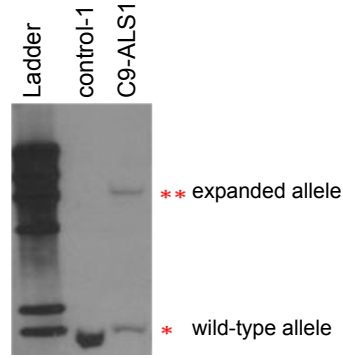

**c**

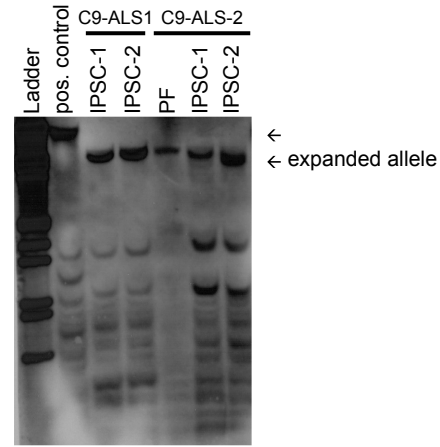

**d**

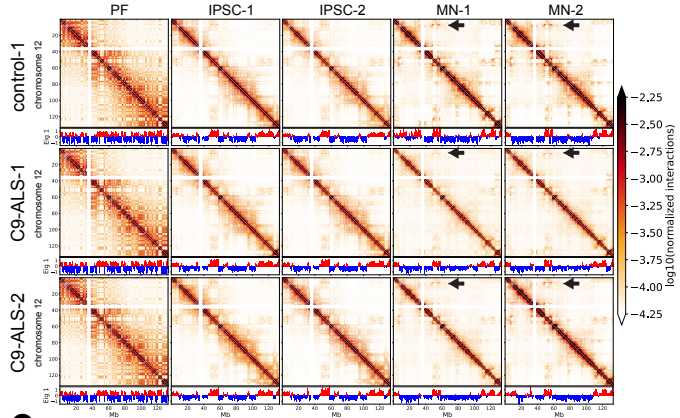

**e**

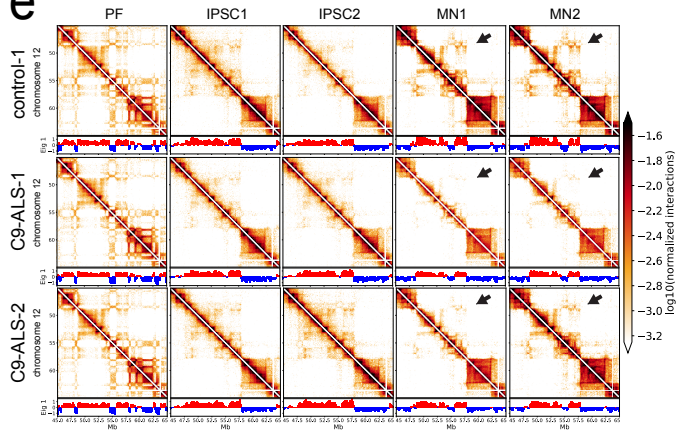

**f**

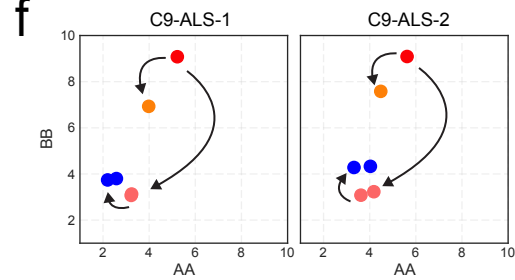

**g**

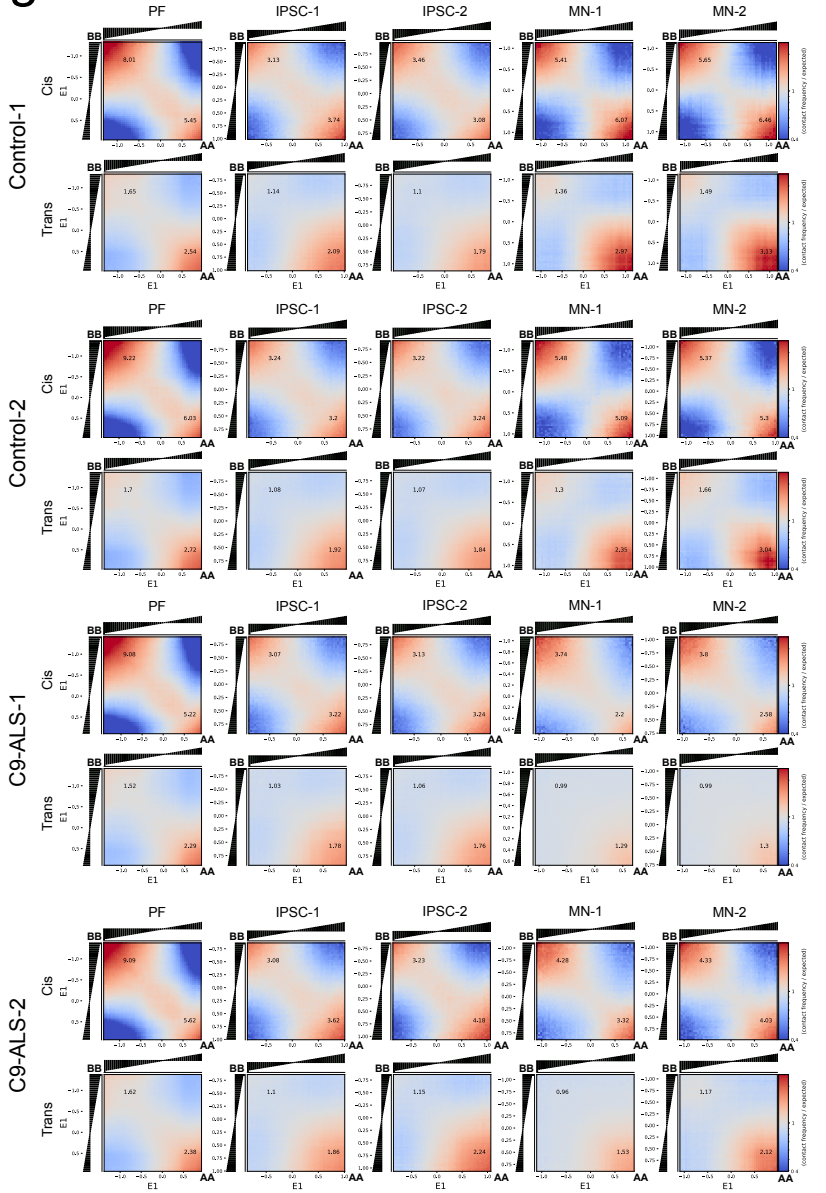

Supplement: Supplement 5 [file media-5.pdf]

h

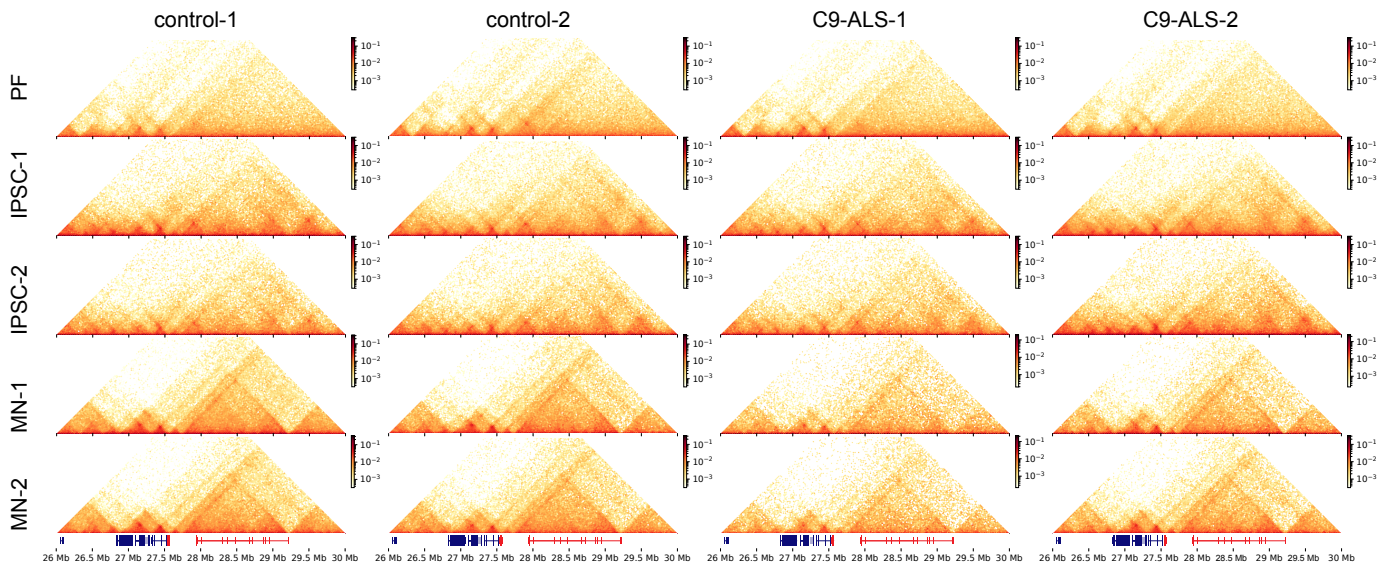

i

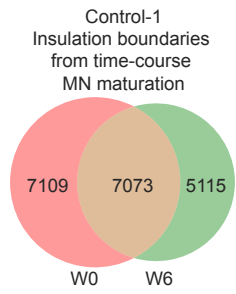

j

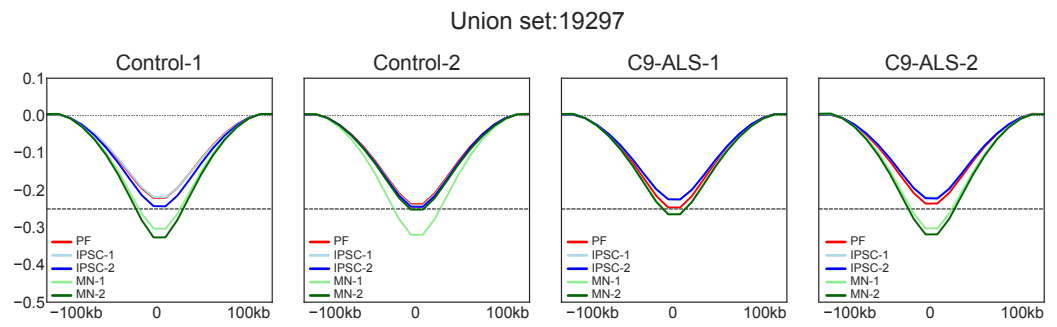

k

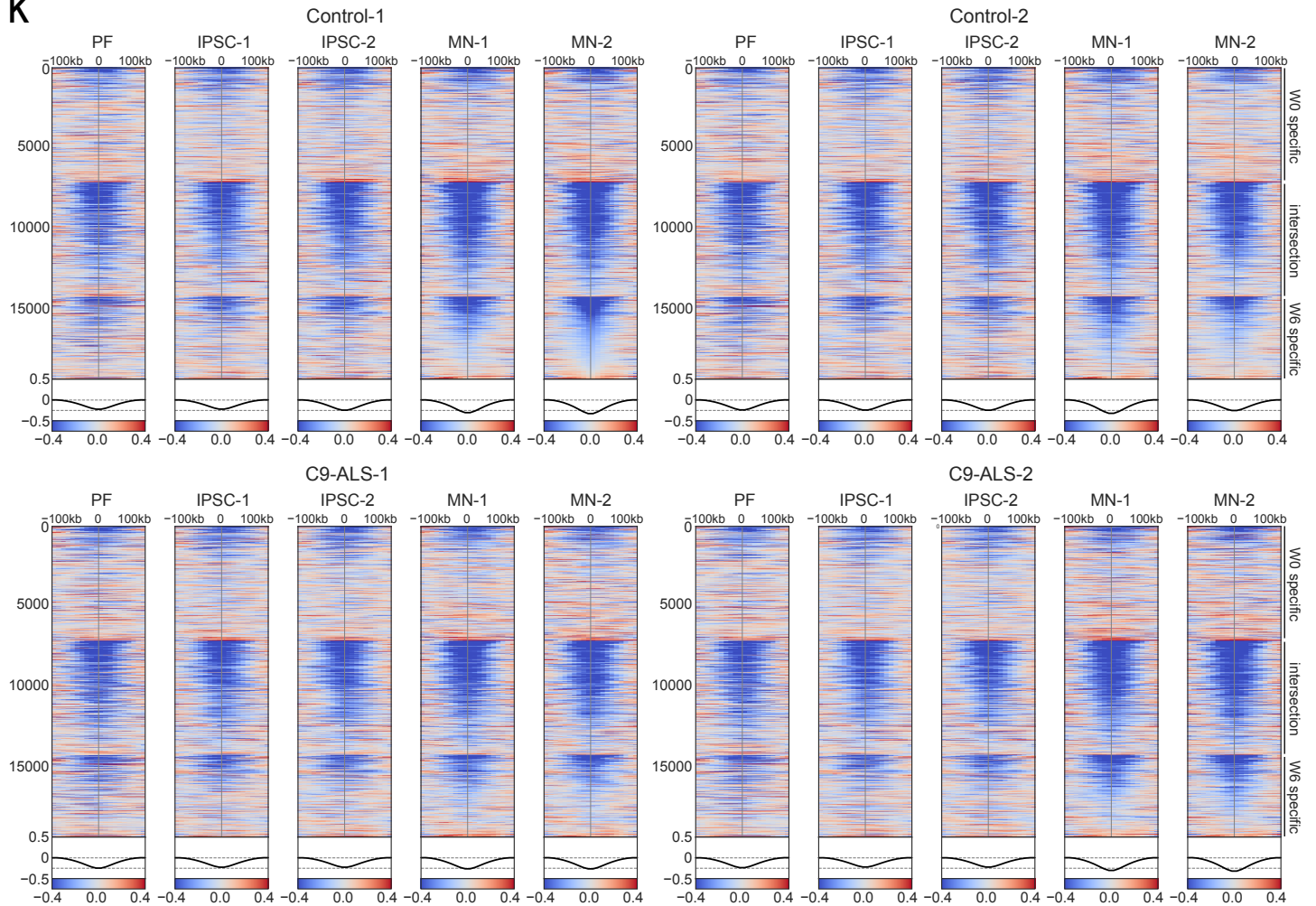

Supplement: Supplement 6 [file media-6.pdf]

# Figure S8

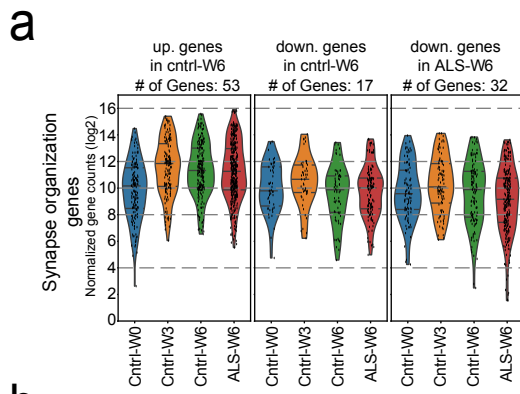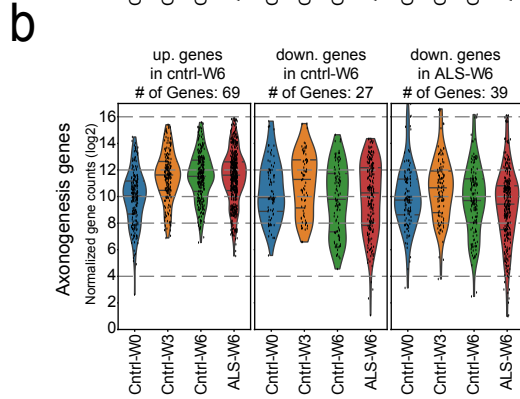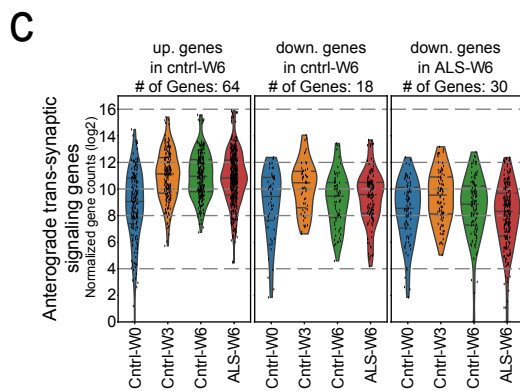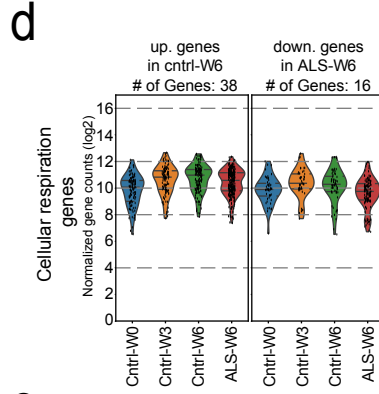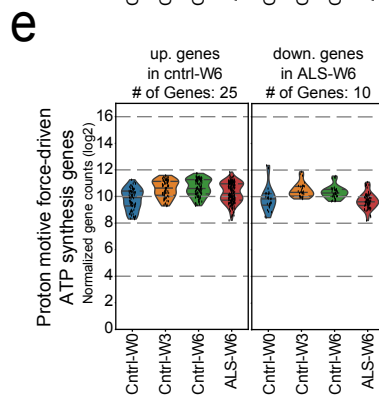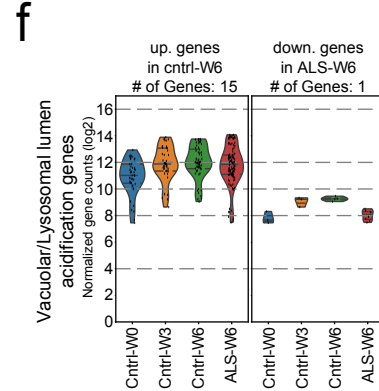

Supplement: Supplement 7 [file media-7.pdf]

Figure S8

g

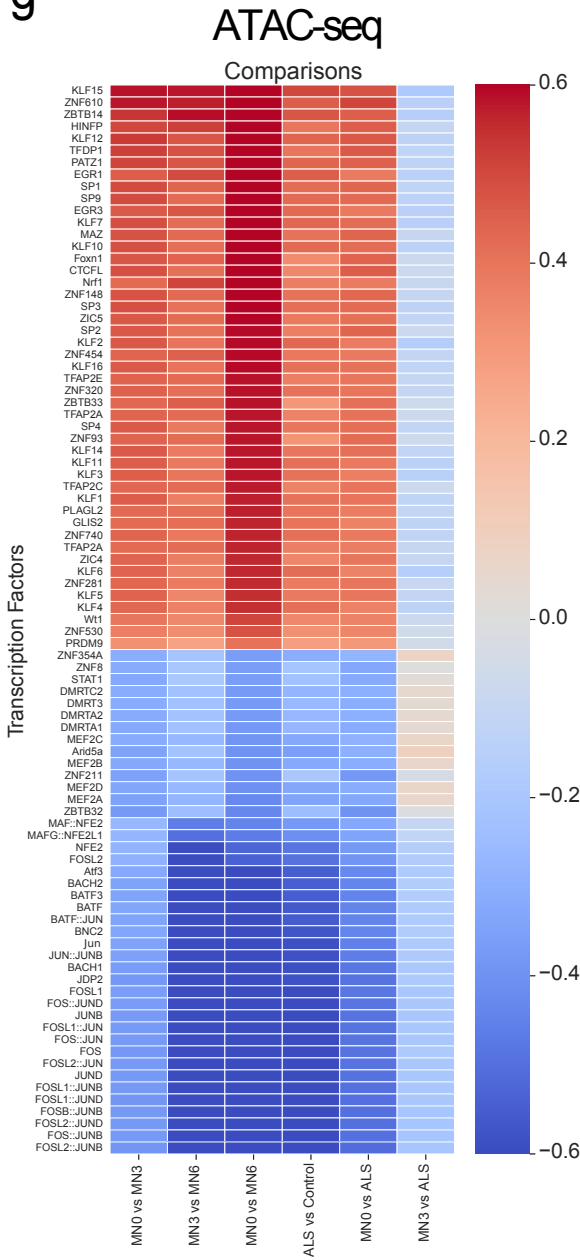

Supplement: Supplement 8 [file media-8.pdf]
