# Supplementary material for: Dynamic changes in chromosome and nuclear architecture during maturation of normal and ALS C9orf72 motor neurons
Source: bioRxiv. 2025 Sep 22:2025.09.22.677835. Preprint. [Version 1] doi: 10.1101/2025.09.22.677835 (PMC12485917; doi:10.1101/2025.09.22.677835)
Supplement: Supplement 9 [file NIHPP2025.09.22.677835v1-supplement-9.pdf]

bioRxiv preprint doi: https://doi.org/10.1101/2025.09.22.677835; this version posted September 22, 2025. The copyright holder for this preprint (which was not certified by peer review) is the author/funder, who has granted bioRxiv a license to display the preprint in perpetuity. It is made available under aCC-BY 4.0 International license.

SUPPLEMENTAL INFORMATION

Materials and Methods

Primary cell lines and cell reprogramming

Two primary normal human dermal fibroblast cells (control-1 and control-2), one male and one female individual were obtained from Coriell institute. ALS patients with C9orf72 HRE primary fibroblast lines were obtained from the repository of the Brown lab (C9-ALS-1 and C9-ALS-2). All primary fibroblasts (PF) were cultured in DMEM, high glucose, GlutaMAXTM supplement with pyruvate (Gibco, cat. no. 10569016) with 10% fetal bovine serum (Sigma, cat. no. F2442) at 37°C in 5% CO2. Cells were passaged every 3-4 days before reaching confluency. For PF Hi-C, cells were harvested at day 4 and day 16. All PF lines were reprogrammed using SeV-based reprogramming kit (CytoTune 2.0 sendai reprogramming kit, life technologies). Protocol is applied as indicated in manufacturer's instructions. Briefly, 1- 2.5 x 105 fibroblast cells were seeded in three wells of a 6-well plate and cultured 2 days to reach 50–65% confluency. SeV reprogramming vectors (CytoTune 2.0, KOS; CytoTune 2.0 hL-Myc; CytoTune 2.0 hKlf4) were added as guided in the protocol. Spent media was replenished every other day until day 7. On day 7 after viral exposure, cells were detached using Trypsin-EDTA (0.25%) (Gibco, cat. no. 25200056), and re-plated on 6-well plates coated with Matrigel (Corning, cat. No. 354277) and maintained in mTeSR1 medium (Stem Cell Technologies) with daily feeding. After 4-5 weeks post-transduction, human iPSC colonies became visible and expanded to be separated from undifferentiated fibroblasts. For each primary fibroblast line that is derived, two IPSC clones were maintained and sent to karyotype analysis for proper chromosome integrity (WiCell).

Maintenance of IPSCs and differentiation of IPSCs into motor neurons

IPSCs were maintained in mTeSR1 medium (Stem Cell Technologies) on 6-well culture plates coated with Matrigel (Corning, cat. no. 354277) at 5% CO2 and 37 °C incubators. IPSCs were incubated in DPBS (Gibco, cat. no. 14190144) containing 1 mM EDTA for few minutes and then dissociated with pipette up and down. Small aggregates of cells with very low density were passaged into new matrigel coated plates containing mTeSR1 medium with 10 µM ROCK inhibitor (Sigma, Y-27632). Fresh mTeSR1 medium was added 24 hours later to remove ROCK inhibitor from the cells. Cells were harvested when they reach 80- 90% confluency for Hi-C and RNA-seq experiments. Motor neuron differentiation was performed as described previously (Klim et al., 2019). In brief, IPSCs were plated on Matrigel-coated culture plates with mTeSR1 medium containing 10 µM ROCK inhibitor. When cells reached 100% confluency, media was changed to differentiation medium (%50 Neurobasal (Gibco, cat. no. 21103049) and 50% DMEM-F12 (Gibco, cat. no. 11320033) containing 1x B-27 supplement (Gibco, cat. no. 17504044), 1x N-2 supplement (Gibco, cat. no. 17502048), 1x GlutaMAX supplement (Gibco, cat. no. 35050079) and 1x MEM Non-Essential Amino Acids (NEAA) Solution (Gibco, cat. no. 11140076)). The following treatments with small molecules were carried out for days 1-6: 10 µM SB-431542 (sigma, cat. no. S4317), 100 nM LDN-193189 (Stemgent/reprocell, cat. no. 04-0074-02), 1 µM retinoic acid (RA) (Sigma, cat. no. R2625) and 1 µM Smoothened agonist (SAG) (Sigma-aldrich, cat. no. 566660); for days 7-14, 5 µM DAPT (Sigma-aldrich, cat. no. D5942), 4 µM SU-5402 (Sigma-aldrich, cat. no. SML0443), 1 µM RA and 1 µM SAG.

bioRxiv preprint doi: https://doi.org/10.1101/2025.09.22.677835; this version posted September 22, 2025. The copyright holder for this preprint (which was not certified by peer review) is the author/funder, who has granted bioRxiv a license to display the preprint in perpetuity. It is made available under aCC-BY 4.0 International license.

Fluorescence-activated cell sorting (FACS) of immature motor neurons

At day 14 after neural differentiation, cultures were dissociated to single cells using StemPro accutase treatment (Gibco, cat. no. A1110501) containing DNAseI (Worthington biochem, cat. no. LK003172) for 30-45 minutes at 37 °C. Gentle pipetting with a 1,000 µl pipetman was used (10–20 up and downs) for complete cell dissociation. Cells were passed through a 40 µm filter and spun down, washed ×1 with DPBS and resuspended in sorting buffer (1x cation-free PBS (Gibco, cat. no. 14190144), 15 mM HEPES (Gibco, cat. no. 15630080), 1% BSA (Sigma, cat. no. A8412-100ML), 1x penicillin- streptomycin (Gibco, cat. no. 15140122), 1 mM EDTA (Invitrogen, cat. no. AM9260G). Single cell suspensions were incubated in sorting buffer containing NCAM (BD Bioscience, cat. no. BDB557919; 1:200 dilution) and EpCAM (BD Bioscience, cat. no. BDB347198; 1:50 dilution) antibodies for 30 minutes and washed with sorting buffer and then resuspended in sorting buffer with DAPI (Invitrogen, cat. no. D3571). The sorted EpCAM-negative NCAM-positive cells were collected and plated onto Poly-L-lysine coated plates (Sigma, P5899) with motor neuron media (Neurobasal medium, 1x N-2 supplement, 1x B-27 supplement, 1x GlutaMax and 1x MEM NEAA solution) with 10 µM ROCK inhibitor and 10 ng per ml of the following neurotrophic factors: glial cell-derived neurotrophic factor (GDNF) (R&D systems, cat. no. 212-GD-010/CF), brain-derived neurotrophic factor (BDNF) (R&D systems, cat. no. 11166-BD-010) and ciliary neurotrophic factor (CNTF) (R&D systems, cat. no. 257-NT-010/CF). Half of the spent media was replenished every 2-3 days. Motor neurons were cultured up to 6 weeks until they were harvested for Hi-C, RNAseq and ATAC-seq.

RNA Fluorescence in situ Hybridization (FISH)

RNA FISH was performed using a 5’ end Cy3-conjugated (G2C4)4 DNA probe to detect sense RNA of the C9orf72 gene. Fibroblasts were fixed in 1x DEPC-treated PBS containing 4% PFA for 15 mins. Fixed cells were washed three times with PBS and were permeabilized with 0.5% triton X-100 (Sigma, cat. no. x100-100ml) in PBS for 10 mins. Then cells were incubated at 56°C in hybridization buffer including 40% formamide (Fisher, cat. no. BP227-500), 2x saline sodium citrate (SSC) buffer, 0.1% Tween-20 and Herring sperm DNA (Promega, cat. no. D1815) for 1 hour. After that cells were incubated with hybridization buffer containing Cy3-conjugated (G2C4)4 DNA probe. Cells were washed twice in pre-warmed wash buffer (40% formamide, 2x SSC, 0.1% Tween-20 (ThermoFisher, cat. no. 85113)) and then in stringency wash buffer (0.2x SSC and 0.1% Tween-20) at 55°C. Samples were then mounted in Prolong Diamond Antifade reagent with DAPI (ThermoFisher, cat. no. P36962) for at least 24 hours.

Southern Blot

8-10 µg genomic DNA (gDNA) was extracted for each sample using gentra puregene blood kit (Qiagen). The gDNA was digested with AluI (NEB, cat. no. R0137L) and DdeI (NEB, cat. no. R0175L) restriction enzymes at 37°C overnight. DNA samples were loaded on 0.8% agarose gel and separated by electrophoresis for 2 hours. Then, the trimmed gel was washed with depurination Buffer (0.25M HCl) for 20 min and rinsed with ddH□O briefly three times. The gel was shaken in denaturation buffer (0.5M NaOH/ 1.5M NaCl) for 40 min and rinsed again with ddH□O briefly three times. Finally, the gel was incubated in neutralization buffer (0.5M Tris-

bioRxiv preprint doi: https://doi.org/10.1101/2025.09.22.677835; this version posted September 22, 2025. The copyright holder for this preprint (which was not certified by peer review) is the author/funder, who has granted bioRxiv a license to display the preprint in perpetuity. It is made available under aCC-BY 4.0 International license.

HCl/ 1.5M NaCl; pH 7.5) for 30 min and rinsed briefly with ddH□O three times. Then, samples were transferred to a positively charged nylon membrane (Roche) using conventional southern blot sandwich method. Transferred gDNA on the nylon membrane was cross-linked by UV (1200J) and incubated with Easy HYB buffer (Roche- Cat no. 11603558001) containing 150µl of Herring Sperm DNA (Promega, cat. no. D1815) (Blocking buffer). The blot was hybridized with a digoxigenin-labeled G2C4 DNA probe in fresh Easy HYB buffer overnight at 55°C. Next day, the blot was firstly washed twice with low stringency wash buffer (2xSSC + 0.1% SDS) at room temperature for 15 min and then washed twice with high stringency wash buffer (0.1x SSC + 0.1% SDS) at 68°C for 30 min. Finally, blot was prepared for detection using DIG wash and block buffer kit (Roche, cat. no. 11585762001). The digoxigenin-labeled probe was detected with anti-digoxigenin antibody and CDP-Star reagent (Roche, cat. no. 12041677001).

Immunoblot analysis

Motor Neuron week 3 and 6 total proteins were extracted using RIPA buffer containing complete mini protease inhibitor cocktail tablets (Roche, cat no. 11836153001). Protein concentration was calculated using Pierce BCA protein assay kit (ThermoFisher, cat no. 23225). 5µg of total protein was loaded onto either 12% or 4-12% Tris-Glycine gels (Invitrogen). Proteins were separated using a mini gel tank (Invitrogen), in 1x TG-SDS running buffer (Invitrogen) at 120 volts for 1.5 hours. Proteins were transferred to nitrocellulose membranes using an iBlot 2 device (Invitrogen) following manufacturers protocols. Membranes were immediately placed into Intercept blocking buffer (LICOR) and incubated at RT for 1 hour. After blocking, the blots were placed into primary antibody solution which consisted of Intercept blocking buffer (LICOR), 0.1% Tween-20 (Fisher), and various combinations of the following primary antibodies; rabbit anti-RAD21 (Abcam, cat no. ab992, 1:1000), rabbit anti-H3 (Abcam, cat no. ab1791, 1:1000), goat anti-βactin (Abcam, cat no. ab8229, 1:3000), rabbit anti-HP1α (Abcam, cat no. ab109028, 1:1000), Rabbit anti-TDP-43 (Proteintech, cat no. 10782-2-ap, 1:1000). Blots were incubated with primary antibody solution overnight at 4°C. The next day, blots were washed 4x with PBS-T, 5 minutes for each wash. The blots were then transferred to secondary antibody solution; intercept blocking buffer (LICOR), 0.1% tween-20 (fisher), and the appropriate Infrared tagged secondary antibodies (LICOR). Blots were incubated in light blocking containers for 1 hour at RT before being washed 4x with PBS-T (5 minutes each), followed by one final wash in PBS for 5 minutes. Blots are stored in PBS in light blocking containers until being visualized on an Odyssey Infrared imager. Densitometry was determined using the Odyssey V3.0 software.

RNA-seq library preparation and data processing

Total RNAs of fibroblast, IPSC and MN lines were extracted using Direct-zol RNA Microprep Kit (Zymo, R2061) with DNAseI digestion. RNA-seq libraries were prepared using KAPA RNA HyperPrep with RiboErase as instructed in manufacturer's protocol (Roche, cat. no. 08098131702). RNA-seq libraries were amplified by 8 PCR cycles using Illumina adapters and primers (TruSeq DNA LT kit Set A 15041757). The libraries were sequenced using 150bp paired end reads on Illumina HiSeq4000.

Fastq reads of RNA-seq libraries were processed using DolphinNext/ViaFoundry platform (Yukselen et al., 2020). Briefly, adapter removal was performed to remove adapter containing sequences from the fastq reads and then sequential mapping was run to remove

bioRxiv preprint doi: https://doi.org/10.1101/2025.09.22.677835; this version posted September 22, 2025. The copyright holder for this preprint (which was not certified by peer review) is the author/funder, who has granted bioRxiv a license to display the preprint in perpetuity. It is made available under aCC-BY 4.0 International license.

rRNAs, miRNAs, snRNAs, piRNAs and tRNAs. RSEM was performed using STAR to align reads to reference transcriptome (Gencode v43) to estimate gene and isoform expression levels. To identify differentially expressed genes, adapter removal and low-quality base trimming were performed with Cutadapt. Gene expression was quantified using Salmon (Patro et al., 2017). DESeq2 (Love et al., 2014) pipeline was run using UMassMed dolphinnext/viafoundry servers (dolphinnext.umassmed.edu/viafoundry.umassmed.edu). TPM (transcript per million) count tables were used in gene expression comparisons among cell-types.

For significant differentially expressed gene selection, the following cut-offs were used: Genes that have less than 100 counts among the samples were removed for the DESeq2 analysis. Furthermore, >1.5 fold-change and p-values<0.05 were applied. For gene set enrichment analysis (GSEA), upregulated and downregulated gene lists were analyzed using Enrichr (Xie et al., 2021), GO Biological Process 2025.

Omni ATAC-seq and analysis

OmniATAC-seq was performed as published previously (Corces et al., 2017). Briefly, motor neurons were washed and lysed while adherent to the plate using 0.1% NP-40, 0.1% Tween-20, 0.01% digitonin, 10 mM Tris-HCl (pH 7.4), 10 mM NaCl and 3 mM MgCl2. Chromatin from approximately 50,000 nuclei was transposed using 10uL Tn5 from the Nextera DNA library prep kit (Illumina, cat. no. FC-121-1030) for 30 min at 37C in the presence of 0.01% digitonin and 0.1% Tween-20. DNA was immediately purified using Qiagen MinElute Kit (Qiagen, cat. no. 28004) and kept at -20°C until PCR amplification. Cycle number for the amplification of the DNA was determined using qPCR. All samples were amplified using less than 8 PCR cycles. Primers were removed from amplified libraries using AMpure XP beads (Beckman Coulter, cat. no. A63881). The libraries were sequenced on illumina HiSeq4000 for 50bp paired end reads.

ATAC-seq data were mapped and processed as described previously (Oomen et al., 2019). Briefly, reads were trimmed from 24 bp and aligned to hg38 using Bowtie2 with maximum mapping length of 2000 bp (Langmead and Salzberg, 2012). Mapped reads were filtered for mapping quality, mitochondrial reads, PCR duplicates and blacklisted. For downstream analysis, each end +5 bp was taken and analysis was continued treating the reads as single end reads. Further downstream processing was performed using BEDtools (Quinlan and Hall, 2010) and HOMER (Heinz et al., 2010).

Footprinting analysis of ATAC-seq data

We used MACS3 (Zhang et al., 2008) to find ATAC-seq peaks of accessibility using default parameters with a shift/extend of -75/+150. The union peak set was merged using bioframe (Open2C et al., 2024). Footprinting analysis was performed using TOBIAS (Bentsen et al., 2020) across the union peak set generated for the “all control MN, all ALS MN, MN-w0, MN-w3, and MN-w6” conditions. First, ATACseq signal was corrected for Tn5 insertion bias using the filtered alignments (ATACorrect). The corrected signal was then used to calculate a continuous footprinting score (ScoreBigwig), determining local regions of decreased accessibility. Finally, mean transcription factor footprinting scores as well as pairwise differential “binding” scores (BINDetect) were determined for 841 conserved vertebrate motifs (Fornes et al., 2020), across the union peak set. From BINDetect scores, bound change values of transcription factors with

bioRxiv preprint doi: https://doi.org/10.1101/2025.09.22.677835; this version posted September 22, 2025. The copyright holder for this preprint (which was not certified by peer review) is the author/funder, who has granted bioRxiv a license to display the preprint in perpetuity. It is made available under aCC-BY 4.0 International license.

significant p-values for MN-w6 vs MN-w0 comparison were extracted as a list. Pairwise bound change scores of the transcription factors listed for MN-w0 vs MN-w3, MN-w3 vs MN-w6, ALS vs control, MN-w0 vs ALS, MN-w3 vs ALS were also extracted and comparisons were plotted as a heatmap. Additionally, log2foldchange values for available genes in the list were also plotted as a heatmap for comparison.

Hi-C library preparation

Hi-C 2.0 was performed as described previously (Belaghzal et al., 2017). 5-10 million cells for each line were fixed with 1% formaldehyde in PBS for exactly 10 minutes at room temperature. 125mM glycine was added and incubated to quench the formaldehyde and terminate the cross-linking reaction for 5 mins at room temperature (RT) and then put on ice for 15 minutes. Cells were scraped from the plates, spun. Supernatant was discarded and then proceeded to cell lysis. 1 ml of ice-cold lysis buffer (10mM Tris-HCl pH8.0, 10mM NaCl, 0.2% Igepal CA630 (Sigma, cat. no. I8896-50ML), 10µL of 10X protease inhibitors (Thermofisher, cat no. 78438)) was added on the fixed cells and put on ice for 15 minutes. Then, cells were lysed with a dounce homogenizer and pestle A (Kimble Kontes cat. no. 885303-0002) by moving up and down 30 times, incubating on ice for one minute followed by 30 more strokes with the pestle. For motor neuron preps, this step was repeated one more time. The suspension was centrifuged for 5 minutes at 2,000g at RT and supernatant was discarded, and the pellets were washed twice with ice-cold 500µL of 1x NEBuffer 3.1. Then the pellet was resuspended in 720µL of 1x NEBuffer 3.1. 38µL of 1% SDS was added to each tube and incubated at 65°C for 10 minutes to solubilize chromatin and then put on ice to cool down. 43µL of 10% Triton X-100 was added to quench SDS. Next, chromatin was digested by adding 400 Units DpnII enzyme (NEB) at 37°C for 16-18 hours digestion within a shaker and then incubated at 65°C for 20 minutes to inactivate the enzyme and then directly put on ice. Biotin fill-in was performed to mark the ends of digested DNA fragments. 60µL of biotin fill-in master mix (1X NEB 3.1, 0.25mM dCTP, 0.25mM dGTP, 0.25mM dTTP, 0.25mM biotin-dATP (ThermoFisher.cat. no. 19524016), DNAvPolymerase I, large Klenow fragment (NEB, cat. no. M0210)) was added to each tube and incubated at 23°C for 4 hours. Biotin filled ends were ligated by adding 665µL of ligation mix (243µL ultra-pure water (Invitrogen, cat. no. 10977015), 240µL of 5X ligation buffer (1.8X) (Invitrogen, cat. no. 46300018), 120µL 10% Triton X-100, 12µL of 10mg/mL BSA and 50µL T4 DNA ligase (Invitrogen, cat. no. 15224090)) and incubated at 16°C for 4 hours. Then, crosslink was reversed by adding 50µL of 10mg/mL proteinase K (Fisher scientific, cat. no. BP1750I-400) and incubated at 65°C for 3 hours. Another 50µL of proteinase K was added and incubated overnight at 65°C. DNA was extracted by adding 1:1 volume of saturated phenol pH 8.0: chloroform (25:24) (Fisher scientific, cat. no. AC327115000), and vortexed for 15 seconds and then transferred to phase-lock tubes and spun at 16,000g for 5 minutes to separated inorganic and organic phases. Ligated DNA was precipitated by adding a volume of 3M sodium acetate pH 5.2 equal to 10% of the initial volume and 2 volumes of ice-cold ~100% ethanol (Fisher scientific, cat. no. BP2818500). Tubes were mixed well and incubated for at least two hours at -80°C. Next, they were centrifuged at 16,000g at 4°C for 30 minutes. The pellet was dissolved in 500µL of 1X Tris-EDTA (TE) (IDT, cat. no. 11-05-01-09) and transferred to a 0.5 ml amicon ultra centrifuge filter (EMD Millipore, cat. no. UFC5030BK). Another 500µL of 1X TE was added to the samples, then, columns were spun at max g for 5 minutes and the flowthrough was discarded. This step was repeated four times. The pellet was dissolved in 50µL of ultra-pure water. 1µL of

bioRxiv preprint doi: https://doi.org/10.1101/2025.09.22.677835; this version posted September 22, 2025. The copyright holder for this preprint (which was not certified by peer review) is the author/funder, who has granted bioRxiv a license to display the preprint in perpetuity. It is made available under aCC-BY 4.0 International license.

10 mg/mL RNAase A was added and incubated at 37°C for 30 minutes. Finally, DNA was quantified by loading on a 1% agarose gel. Hi-C libraries were treated with T4 DNA polymerase to remove unbiotinylated DNA fragment ends. Up to 5µg DNA of Hi-C library was added to a 50µL reaction tube containing 5µL of 10X NEBuffer 3.1, 0.025mM dATP, 0.025mM dGTP and 15U T4 DNA polymerase (NEB, cat. no. M0203L). Each tube was incubated at 20°C for 4 hours, and then inactivated at 75°C for 20 minutes. Appropriate volume of 1X TE was added to make it 130µL. DNA fragments were sheared to a size of ~200bp using a covaris instrument (Duty Factor 20%, Cycles per Burst 200, peak power 50, average power 17.5 and process time 180 sec). Size selection was applied to enrich DNA fragments between 100-300bp using ampure XP fractionation (Beckman Coulter, cat. no. A63881) and the pellet was eluted with 50µL of ultra-pure water. The quality of DNA fragments was checked on a 2% agarose gel. Next, end repair was performed using 45µL of Hi-C samples transferred to each PCR tube, and 25µL of the end-repair mix containing 3.5X NEB ligation buffer (NEB, cat. no. B0202S), 17.5mM dNTP mix, 7.5U T4 DNA polymerase (NEB, cat. no. M0203L), 25U T4 polynucleotide kinase, 2.5U Klenow polymerase Polymerase I, was added and incubated at 37°C for 30 minutes. Then tubes were additionally incubated at 75°C for 20 minutes to inactivate the enzymes. 50µL of MyOne streptavidin C1 bead mix (ThermoFisher, cat. no. 65001) was transferred to a 1.5mL tube. The beads were washed twice by adding 400µL of TWB (5mM Tris-HCl pH=8, 0.5mM EDTA, 1M NaCl, 0.05% Tween20) and incubated for 3 minutes at RT. Then, beads were resuspended in 400µL of 2X binding buffer (10mM Tris-HCl pH=8, 1mM EDTA, 2M NaCl) and mixed with 400µL of DNA fragments from the previous step. The mixture was incubated for 15 minutes at RT with rotation. Beads with bound DNA were washed with 400µL of 1X binding buffer and then washed once with 100µL of 1X NEB2.1. Finally, beads containing DNA were resuspended in 41µL of 1x NEB2.1. 9µL of A-tailing mix (5µL of NEB buffer 2.1, 5µL of 1mM dATP, 3U Klenow exo (NEB, cat. no. M0212S)) was added on top of 41µL of beads. The reaction was incubated at 37°C for 30 minutes, then at 65°C for 20 minutes, and then cooled to 4°C. The beads containing bound DNA were washed twice with 100µL of 1X T4 DNA ligase buffer and then resuspended in 35µL 1X T4 DNA ligase buffer. For final step of library preparation, PCR amplifications, TruSeq DNA LT kit Set A (Illumina, cat. no. 15041757) was used. Three different PCR cycles (6-8-10) were tested to pick best amplification cycle for each sample. 6-8 PCR cycles was performed for final library preparation using primer mix and Master Mix of TruSeq DNA LT kit Set A. Final library products were cleaned up using Ampure XP beads and sequenced using 50bp paired end reads on an Illumina HiSeq4000 instrument.

Hi-C data processing

Distiller (https://github.com/mirnylab/distiller-nf) pipeline was used to process Hi- C fastq files. First, fastq reads were mapped to hg38 using bwa mem. Second, mapped reads were parsed and classified using the pairtools package (https://github.com/mirnylab/pairtools) to get pairs files. PCR duplicates were removed, and pairs were filtered using mapping quality scores (MAPQ > 30). Data were binned into 1, 2, 5, 10, 25, 50, 100, 250, 500 and 1000kb resolutions. Finally multiresolution cooler files were created using the cooler package (https://github.com/mirnylab/cooler.git). Contact matrices were normalized using the iterative correction procedure published previously (Imakaev et al., 2012).

Cis and Trans Ratio

bioRxiv preprint doi: https://doi.org/10.1101/2025.09.22.677835; this version posted September 22, 2025. The copyright holder for this preprint (which was not certified by peer review) is the author/funder, who has granted bioRxiv a license to display the preprint in perpetuity. It is made available under aCC-BY 4.0 International license.

Cis and trans percentages were calculated by dividing the total interactions between chromosomes with the sum of interactions within and between chromosomes. The numbers of interactions captured within and between chromosomes were obtained from pairtools.

Compartment Analysis

A and B compartments were assigned using an eigenvector decomposition procedure implemented in the cooltools package. Eigenvector decomposition was performed on observed-over-expected cis contact matrices at 25 kb and 100kb bin size for every chromosome. The first eigenvectors (EV1/Eig1) positively correlated with the gene density were used to call A or B compartment identity for each bin. Compartmentalization strengths were calculated using saddle-plots as implemented in cooltools version 0.5.4 (Imakaev et al., 2012). Briefly, saddle plots were generated by sorting the eigenvector values from lowest to highest (B to A). Then, sorted maps were normalized with their expected interaction frequencies. The upper left corner of the interaction matrix represents the strongest B-B interactions and the lower right for strongest A-A interactions. Upper right and lower left are for B-A and A-B respectively. To quantify and compare saddle plots of different samples, the strongest 20% of BB and strongest 20% of AA interactions were taken and divided them by the sum of AB and BA (AA)/(AB+BA) and (BB)/(AB+BA).

For generating centromere-telomere plots (Figure 2H and 7D), the saddles were piled up using generated centromere and telomere CT track. Centromere-telomere tracks were generated for every chromosome at 100kb bin size, by assigning values from 0 to 1 for p arm, and 1 to 2 for q arm with 1 representing centromeric regions, and 0 and 2 the telomeric regions. Cooltools-saddle function of trans pile was used to generate the pileups for chromosomes 1 to 22.

For arm specific telomeric interaction plots (Figure 5h and 7E), telomeric interactions were obtained from 250kb matrices. First four 250kb bins of each telomeric end with valid balance weight score (1 Mb) was used to obtain total trans and cis telomeric interactions from balanced cooler matrices for all autosomal chromosomes. First diagonal scores indicate cis telomeric end interaction value for each chromosome. Other squares with 4 sub squares indicate trans telomeric arms' interaction scores between chromosome pairs.

Insulation score analysis

insulation scores were calculated using cooltools (https://github.com/open2c/cooltools/blob/master/cooltools/cli/diamond_insulation.py) as implemented from Crane et al (Crane et al., 2015). The insulation analyses were done on 10kb binned data with a 100kb window size. First two diagonals were excluded for insulation score calculations. Strong boundaries were defined by the boundary strength values greater than or equal to 0.1 for comparison of week 6 and week 0 samples. Genomic bin that has "nan" values in at least 1 bin out of 5 flanking right or left bins were removed. The regions that are common and have an overlap of 5kb were considered as intersected regions. For generating stack-ups, bbi.stackup() function was used, with 100kb flanking regions. The compartments were called at 25kb resolution and insulation at 10kb with a 100kb sliding window. The regions were arranged based on compartment change status across the middle bin with regions sorted in descending

bioRxiv preprint doi: https://doi.org/10.1101/2025.09.22.677835; this version posted September 22, 2025. The copyright holder for this preprint (which was not certified by peer review) is the author/funder, who has granted bioRxiv a license to display the preprint in perpetuity. It is made available under aCC-BY 4.0 International license.

order of EV value as a second key of sorting. The insulation stack-ups were normalized by subtracting the mean of first three and last three bins of each row from itself.

bioRxiv preprint doi: https://doi.org/10.1101/2025.09.22.677835; this version posted September 22, 2025. The copyright holder for this preprint (which was not certified by peer review) is the author/funder, who has granted bioRxiv a license to display the preprint in perpetuity. It is made available under aCC-BY 4.0 International license.

Supplementary figures

Figure S1 (associated with Figure 1). Generating induced pluripotent stem cells from primary fibroblasts

a. Schematic view of the study design.
b. Schematic view of IPSC generation from primary fibroblast using CytoTune iPS 2.0 Sendai Reprogramming Kit.
c. Formation of Embryoid bodies (EB) to generate three germ lines from IPSC clones. TUBB3 (Tuj1): βIII-tubulin (Neural, ectodermal); SMA: Smooth muscle Actin, mesodermal; SOX17: SRY (sex determining region Y)-box 17 (Vascular, endodermal).

Figure S2 (associated with Figure 2). Chromatin architecture of cell type–specific genes in PFs, iPSCs, and MNs.

Hi-C contact matrices at 25 kb resolution with A/B compartment tracks (first eigenvector, EV1) are shown for PFs, iPSCs, and MNs (columns). Each heatmap is centered on the gene of interest, with ±2 Mb of flanking regions. (a) COL1A2 (PF), SOX2 (iPSC), MNX1 and MAP2 (MN); (b)

bioRxiv preprint doi: https://doi.org/10.1101/2025.09.22.677835; this version posted September 22, 2025. The copyright holder for this preprint (which was not certified by peer review) is the author/funder, who has granted bioRxiv a license to display the preprint in perpetuity. It is made available under aCC-BY 4.0 International license.

ANXA2 (PF), ESRG (iPSC), NRG1 and FOXP1 (MN); (c) COL1A1 (PF), L1TD1 (iPSC), NEFH and PRPH (MN); (d) GFAP, S100B, TMEM119, and AIF1 (glial markers). Top panels display GENCODE gene annotations for each region.

Figure S6 (associated with Figure 6). Analysis of TAD boundaries during maturation. Boundary strength distribution of insulation scores sorted by log 10 of their boundary strengths in EP-W0 (Total:48366 Strong:17148), MN-W0 (Total:47012 Strong:16211), MN-W3 (Total:36689 Strong:14521) and MN-W6 (Total:33008 Strong:13599) (strong boundary > log10 -1) Insulation scores and boundary strength are calculated at 10 kb resolution with 100 kb window size.

Figure S7 (associated with Figure 7). Characterization of the *C9orf72* HRE mutation in ALS patients and chromatin architecture in C9-ALS PFs, iPSCs, and MN cells compared to control cells.

a. RNA-fluorescence in situ hybridization (RNA-FISH) in primary fibroblasts of control-1, C9-ALS-1 and C9-ALS-2 individuals using a Cy3 labeled $(G2C4)_4$ DNA probe. White arrows indicate $(G4C2)_n$ containing RNA foci. Blue: DAPI; Red: Cy3-conjugated $(G2C4)_4$ DNA probe.
b. Southern blot analysis of fibroblasts from control-1 and C9-ALS-1. Repeat-containing fragments were detected using a region-specific, 5′ DIG-labeled PCR-amplified probe. The expanded allele shows a repeat size of approximately 800–1000.
c. Southern Blot analysis of IPSCs and fibroblasts obtained from C9-ALS-1 and C9-ALS-2 patients. Repeat expansions were detected using a 5’DIG-$(G4C2)_5$-DIG-3’ DNA probe revealing a ~5-6 kb band corresponding to an expanded repeat size of 800–1000. Positive control: ALS patient with a known *C9orf72* HRE.
d. Hi-C contact matrices of chromosome 12 interactions with A/B compartment calls (100k bin size) in PFs, two IPSCs and two MNs from control-1, C9-ALS-1 and C9-ALS-2, individuals (Eig1= Eigenvalue 1).
e. Zoomed-in Hi-C contact matrices showing compartmental and TAD interactions in the 45–65 Mb region of chromosome 12 (100 kb bin size) for PFs, two iPSCs, and two MNs from control-1, C9-ALS-1, and C9-ALS-2 individuals (Eig1 = first eigenvector).
f. Quantification of compartment strengths (AA and BB) using cis-interactions derived from saddle plots of PFs, iPSCs, and MNs from C9-ALS-1 and C9-ALS-2.
g. Genome-wide cis and trans saddle plots of chromatin interactions in PFs, IPSCs and MNs from control-1, control-2, C9-ALS-1 and C9-ALS-2 individuals.
h. Hi-C contact matrices (25 kb bin size) focused on the 26–30 Mb region of chromosome 9 in PFs, two iPSCs, and two MNs from control-1, control-2, C9-ALS-1, and C9-ALS-2. Bottom panels display GENCODE gene annotations for each region.
i. Venn diagram showing the overlap of strong chromatin boundaries between week 0 and week 6 as derived from Figure 6G. Boundaries include W0-specific (n = 7109), shared (W0 & W6; n = 7073), and W6-specific (n = 5115) sets.
j. Aggregated insulation score pileups of strong boundaries (union from panel i) across PFs, iPSCs, and MNs from control-1, control-2, C9-ALS-1, and C9-ALS-2 individuals.

bioRxiv preprint doi: https://doi.org/10.1101/2025.09.22.677835; this version posted September 22, 2025. The copyright holder for this preprint (which was not certified by peer review) is the author/funder, who has granted bioRxiv a license to display the preprint in perpetuity. It is made available under aCC-BY 4.0 International license.

k. Stack-up plots of insulation scores detected at the strong boundaries with flanking 100kb from each side for control-1, control-2 and C9-ALS-1 and C9-ALS-2 individuals. W0-specific, intersection, and W6-specific strong boundaries detected in figure S6I are used.

Figure S8 (associated with Figure 8). Transcriptome and genome accessibility impacts of C9orf72 HRE Mutation in ALS Motor Neurons

a. Expression levels of genes associated with the synapse organization biological process were plotted across four conditions: cntrl-W0, cntrl-W3, cntrl-W6, and ALS-W6. The panels represent three comparisons. Left panel: up. genes in cntrl-W6 vs cntrl-W0 (# of Genes: 53); mid panel: down. genes in cntrl-W6 vs cntrl-W3 (# of Genes: 17); right panel: down. genes in ALS-W6 vs cntrl-W3 (# of Genes: 32).
b. Expression levels of genes associated with the axonogenesis biological process were plotted across four conditions: cntrl-W0, cntrl-W3, cntrl-W6, and ALS-W6. The panels represent three comparisons. Left panel: up. genes in cntrl-W6 vs cntrl-W0 (# of Genes: 69); mid panel: down. genes in cntrl-W6 vs cntrl-W3 (# of Genes: 27); right panel: down. genes in ALS-W6 vs cntrl-W3 (# of Genes: 39).
c. Expression levels of genes associated with the Anterograde trans-synaptic signaling biological process were plotted across four conditions: cntrl-W0, cntrl-W3, cntrl-W6, and ALS-W6. The panels represent three comparisons. Left panel: up. genes in cntrl-W6 vs cntrl-W0 (# of Genes: 64); mid panel: down. genes in cntrl-W6 vs cntrl-W3 (# of Genes: 18); right panel: down. genes in ALS-W6 vs cntrl-W3 (# of Genes: 30).
d. Expression levels of genes associated with the cellular respiration biological process were plotted across four conditions: cntrl-W0, cntrl-W3, cntrl-W6, and ALS-W6. The panels represent two comparisons. Left panel: up. genes in cntrl-W6 vs cntrl-W0 (# of Genes: 38); right panel: down. genes in ALS-W6 vs cntrl-W3 (# of Genes: 16)
e. Expression levels of genes associated with the proton motive force-driven ATP synthesis biological process were plotted across four conditions: cntrl-W0, cntrl-W3, cntrl-W6, and ALS-W6. The panels represent two comparisons. Left panel: up. genes in cntrl-W6 vs cntrl-W0 (# of Genes: 25); right panel: down. genes in ALS-W6 vs cntrl-W3 (# of Genes: 10)
f. Expression levels of genes associated with the vacuolar/lysosomal lumen acidification biological process were plotted across four conditions: cntrl-W0, cntrl-W3, cntrl-W6, and ALS-W6. The panels represent two comparisons. Left panel: up. genes in cntrl-W6 vs cntrl-W0 (# of Genes: 15); right panel: down. genes in ALS-W6 vs cntrl-W3 (# of Genes: 1)
g. Heatmap displaying the differential binding scores of transcription factors (TFs) with the most significantly upregulated and downregulated binding activity. Genome-wide TF binding dynamics were analyzed using footprinting analysis of ATAC-seq data from control MNs (W0, W3, and W6) and C9-ALS MNs (W6). Differential binding scores for each TF were calculated using TOBIAS.
